# Active porous ZIF-8 based hybrid catalysts for solvent-free reactions

**DOI:** 10.3389/fchem.2026.1894924

**Published:** 2026-09-17

**Authors:** César D. Sampaio, Luís Dias, Isabel Santos-Vieira, Eulália Pereira, Luís Cunha-Silva, Fátima Mirante, Salete S. Balula

**Affiliations:** 1 LAQV / REQUIMTE, Department of Chemistry and Biochemistry, Faculty of Sciences - University of Porto, Porto, Portugal; 2 CICECO – Aveiro Institute of Materials, Department of Chemistry, University of Aveiro, Aveiro, Portugal; 3 LNEG - Laboratório Nacional de Energia e Geologia IP, Lisboa, Portugal; 4 LAQV / REQUIMTE, Department of Chemistry, Nova School of Science and Technology, Nova University of Lisbon, Monte daCaparica, Portugal

**Keywords:** aminolysis, epoxide ring-opening, heterogeneous catalysis, polyoxometalates, porous coordination polymers

## Abstract

Hybrid heterogeneous polymeric catalysts were prepared by distinct methodologies to evaluate their catalytic ability, stability and recycling capacity to promote the ring-opening reaction of styrene oxide with aniline resulting as main product the 2-Amino-1,2-diphenylethanol. These composites were based on porous metal–organic frameworks (MOFs), specifically the zinc-based ZIF-8, activated catalytically by the incorporation of Keggin-type phosphomolybdic acid (PMo_12_). The resulting composite denoted as PMo_12_@ZIF-8 was prepared via impregnation and *in-situ* methods (PMo_12_ loadings of 0.149 and 0.194 mmol/g were obtained via *in-situ* and impregnation procedures). Both materials were characterized by FTIR-ATR, PXRD, SEM, EDS, and ICP-OES. The catalytic performance evaluation revealed that both composites exhibited high conversion rates under a sustainable solvent-free system (98% of conversion was achieved after 24 h), and high selectivity toward 2-Amino-1,2-diphenylethanol, along with notable robustness and recyclability.

## 1 Introduction

Metal–organic frameworks (MOFs) are crystalline coordination polymers with high porosity, large surface area, and flexibility (Ahamad et al., 2020; Kitagawa et al., 2004). MOFs exhibit a high degree of controllability over their structures, making them suitable for various applications such as gas storage, separation, sensing, drug delivery, medical applications, and heterogeneous catalysis (Ali Akbar Razavi and Morsali, 2019; Farrusseng et al., 2009; Păun et al., 2023; Xie et al., 2022). The use of MOFs as heterogeneous catalysts has been one of the most promising applications, along with its rapid development in the last 20 years (Jiao et al., 2018). Due to their exceptional structural tunability, which allows the incorporation of diverse catalytic sites, such as metal centers and functional organic groups within a single framework, porous MOFs have gained considerable attention as versatile heterogeneous catalysts for a broad range of chemical transformations (Chughtai et al., 2015; Ferreira et al., 2024; Liu et al., 2014). In particular, Zeolitic Imidazolate Frameworks (ZIFs) have garnered considerable interest as a genre of MOF, comprising imidazolate anions linked to tetrahedral metal cations such as Zn^2+^, Co^2+^, and occasionally In^3+^ (Park et al., 2006). ZIFs are crystalline materials with porous nature, which have been extensively researched due to their exceptional thermal, chemical, and mechanical stability. Consequently, they present themselves as compelling candidates for various applications, including gas adsorption, separation, and catalysis (Thirumalaikumar, 2022). In this work ZIF-8 MOF structure was selected by its suitable morphology and high stability to accommodate the acid active center phosphomolybdic acid (abbreviated as PMo_12_).

The most effective approach for producing β-aminoalcohols relies on the ring-opening of epoxides via interaction with amines. β-Amino alcohols are usually synthesized by the direct aminolysis of epoxides. This process is favored in the presence of Lewis acids, as well as Bronsted acids and bases (Barbosa et al., 2017). However, this reaction often involves a large excess of amines and high temperatures; thus, to promote useful modifications in habitual procedures, homogeneous catalysts have been widely applied (Danafar and Yadollahi, 2009; Aramesh et al., 2013). Therefore, there is great interest in studying the opening reactions of epoxy ring (Danafar and Yadollahi, 2009; Mohseni et al., 2014; Thirumalaikumar, 2022). Although significant progress has been made in the development of this type of reactions of epoxides, most homogeneous catalysts reported to date remain costly, inefficient, toxic, and prone to instability. Nevertheless, a few systems have demonstrated promising results, achieving high efficiency in the aminolysis of various epoxides under mild reaction conditions. Various types of materials have been used in the heterogenization of catalytically active species, resulting in efficient heterogeneous catalyst-based materials (Becker and Wandelt, 2018; Bavykina et al.,2020). To date there are only a few reports on the use of MOF based composites incorporating polyoxometalates (POMs), i.e. POM@MOFs as catalysts in the ring-opening reaction of cycloalkene oxide (Barbosa et al., 2017; Julião et al., 2017; Wee et al., 2014). MOFs can be utilized in multiple ways to enhance their catalytic potential. One-way active centers such as metal complexes nanoparticles, POMs, and other compounds such as quaternary ammonium, have the possibility to be immobilized within the framework MOF structure due to the large cavities present in MOFs along with their inherent flexibility (Bavykina et al., 2020; Wang, 2022).

Researchers have been highly interested in studying polyoxometalates (POMs) due to their remarkable variety of metals that can be incorporated, as well as their structural versatility. One of the extensively studied and widely utilized structures among all POMs is the Keggin-type POMs. Despite their promising catalytic properties, POMs suffer from severe drawbacks when used in homogeneous systems, including difficult separation, lack of reuse, and potential environmental impact due to the occurrence of leaching into reaction media (Julião et al., 2017). To overcome these limitations, the development of heterogeneous hybrid catalysts via the encapsulation or immobilization of these active species onto solid supports has gained increasing attention. In this context, MOFs, particularly ZIF-type materials, represent an ideal platform for heterogenization due to their chemical tunability, porosity, and ability to host a wide range of functional guest molecules (Thirumalaikumar, 2022). Recent studies have shown that POM@MOF based composites exhibit enhanced acidity and redox activity (Abdelkader-Fernández et al., 2022; Julião et al., 2017). Therefore, combining POMs within a ZIF scaffold may produce synergistic catalytic effects, enhance reactivity while maintaining catalyst stability and recyclability (Abdelkader-Fernández et al., 2022; Faria et al., 2025; Faria et al., 2026; Gao et al., 2023; Silva et al., 2022). However, studies exploring the cooperative role of POM immobilized in the same MOF structure remain limited, and their catalytic behavior in nucleophilic epoxide ring-opening reactions is still poorly understood.

In this work, the preparation of porous materials capable of effectively immobilizing the polyoxometalate (phosphomolybdic acid, PMo_12_) within the cavities of the ZIF-8 framework is presented. Two different immobilization strategies were investigated: impregnation and *in-situ* incorporation. The prepared ZIF-8 based composites were tested in a reaction base in the ring-opening reaction of styrene oxide (ROSO) with aniline. This is a highly interesting catalytic reaction to obtaining valuable products with high industrial interest.

## 2 Materials and methods

### 2.1 Reagents and products

For the synthesis of ZIF-8 and ZIF-8 based composites, were used zinc nitrate hexahydrate (Zn(NO_3_)_2_∙6H_2_O, Sigma-Aldrich, 99%), 2-methylimidazole (C_4_H_6_N_2_, Sigma-Aldrich, 99%), methanol (CH_3_OH, Fisher Scientific, 99%), phosphomolybdic acid (H_3_PMo_12_O_40_∙xH_2_O, abbreviated as PMo_12_, Merck), ethanol (C_2_H_6_O, Honeywell, 99.8%), and tetrabutylammonium bromide (N(C_4_H_9_)_4_Br, Sigma-Aldrich, 99%). In the catalytic studies of ring-opening styrene oxide with aniline (ROSO) reactions, were used styrene oxide (C_8_H_8_O, abbreviated as SO, Sigma-Aldrich, 97%), aniline (C_6_H_7_N, Sigma-Aldrich, 99%), ethanol (C_2_H_6_O, Honeywell, 99%). All chemicals were not subjected to any further purification after synthesis.

### 2.2 Characterization methods

The Attenuated Total Reflectance-Fourier Transform Infrared (FTIR-ATR) Spectroscopy experiments were conducted employing a PerkinElmer Spectrum BX instrument. The measurements were carried out over the range of 400–4000 cm^−1^, with a total of 64 scans and a resolution set at 1 cm^−1^. The results obtained for transmittance values were presented in arbitrary units (a.u.). The analysis of the sample using Powder X-ray diffraction (PXRD) was conducted at room temperature employing an Empyrean PANalytical diffractometer. This diffractometer utilized a radiation source of Cu with a λ = 1.5405980 Å and operated in the Bragg-Brentano configuration. To obtain the diffractograms, measurements were taken within a range spanning from 3° to 50°. The intensities displayed on all diffractograms were given in arbitrary units (a.u.). The *Centro de Investigação em Materiais Cerâmicos e Compósitos* (CICECO), situated at the University of Aveiro (UA), performed this analysis. The Scanning Electron Microscope, along with Energy Dispersive X-Ray Microanalysis (SEM) and Electron Backscattered Diffraction analysis (EDS), were conducted using the FEI Quanta 400FEG ESEM and EDAX Genesis X4M instruments, operating at a voltage of 15 kV. To enhance sample conductivity, all samples were coated with a thin Au/Pd film using the SP IModule Sputter Coater equipment for a duration of 80 s at an applied current of 12 mA. ^31^P NMR spectra were obtained using a Nuclear BRUKER AVANCE III 400 MHz, 9.4 Tesla digital spectrometer (Bruker, Billerica, MA, United States of America). All analyses were performed in the *Centro de Materias da Universidade do Porto* in *Faculdade de Ciências da Universidade do Porto*. The quantification of molybdenum and bromine were determined using Inductively Coupled Plasma Optical Emission Spectroscopy (ICP-OES) at the University of Santiago de Compostela. These analyses were conducted on an Agilent 5,900 instrument equipped with a plasma source Xerador RF operating at a frequency of 40 MHz. The catalytic reactions were monitored using Gas Chromatography in combination with Flame Ionization Detection (GC-FID) on a Scion 8300 Chromatographer. Hydrogen was used as the carrier gas, flowing at a rate of 55 cm^3^ s^−1^. The analysis used fused silica SPB-5 capillary columns, which had dimensions of 30 m length, diameter of 0.25 mm, and a film thickness of 0.25 μm. The Gas Chromatography coupled with mass spectroscopy analysis (GC-MS) used a Hewlett Packard 5890 chromatograph with a Mass Selective Detector MSD series II. Helium gas was used as the carrier in the analysis, flowing at a rate of 35 cm^−1^. The fused silica SPB-5 capillary columns were employed with dimensions measuring 30 m in length, 0.25 mm in diameter, and a film thickness of 0.25 μm N_2_ adsorption-desorption isotherms at − 196 °C were obtained using a Quantachrome NOVA 4200e equipment, with NOVA win software used for data processing. Samples were degassed at 150 °C prior to analysis, and micropore volume and BET surface area were calculated using -t and BET methods, respectively.

### 2.3 Preparations of the materials

#### 2.3.1 ZIF-8

The MOF materials ZIF-8 was synthesized using the method previously described in the literature (Hu et al., 2022). In this procedure, a solution of 1.02 g of Zn(NO_3_)_2_∙6H_2_O in 25 mL of methanol was stirred for a period of 30 min. Subsequently, a solution containing 1.13 g of 2-methylimidazole in 25 mL of methanol was added to the mixture resulting in the formation of a white suspension. The suspension was then left stirring for approximately 2.5 h. The obtained white solid was collected from the suspension through centrifugation which was repeated three times. The isolated materials were subsequently dried at a temperature of 120 °C under a pressure of 175 mBar for about 19 h.

#### 2.3.2 PMo_12_@ZIF-8_is by in-situ approach

The synthesis of this composite material by *In-situ* (is) approach was adapted from the literature (Thirumalaikumar, 2022). A solution of 0.653 g of Zn(NO_3_)_2_∙6H_2_O in 25 mL of methanol was stirred for 15 min, while a solution of 100 mg of PMo_12_ in 5 mL of methanol was stirred for the same amount of time, the POM solution was added to the Zn(NO_3_)_2_∙6H_2_O solution and left to stir for another 15 min. Then a third solution of 1.63 g of 2-methylimidazole in 25 mL of methanol was dropwise added and was stirred for an additional 2.5 h at room temperature. The obtained solid was isolated by centrifugation and washed 5 times with methanol and dried at 60 °C and 120 mBar for 16 h.

#### 2.3.3 PMo_12_@ZIF-8_imp by impregnation approach

The following material was synthesized by an impregnation approach (imp) adapted from the literature (Fernandes et al., 2022). A solution of 100 mg of PMo_12_ and 150 mg of ZIF-8, previously synthesized and dried, were mixed in 10 mL of ethanol and left to stir for 4 days. Subsequently, the mixture was centrifugated, and the obtained solid material was washed 5 times with ethanol and dried at 60 °C and 120 mBar for 16 h.

### 2.4 Catalytic studies

The catalytic efficiency of the previously prepared materials was evaluated in a Ring-Opening reaction of Styrene Oxide with aniline (ROSO). The support and composite materials, ZIF-8, PMo_12_@ZIF-8 prepared by Impregnation Approach (PMo_12_@ZIF-8_imp) and *In-situ* Approach (PMo_12_@ZIF-8_is) were used to catalyze ROSO catalytic reaction. The catalytic investigation of ROSO was conducted according to the methodology outlined in the literature (Julião et al., 2017), (Barbosa et al., 2017). The catalytic reactions were managed in a borosilicate reactor with a volume of 5 mL. The reactor was sealed and placed inside a paraffin bath, which was continuously stirred at a temperature of 70 °C. To initiate the reaction, 127 μL (1.1 mmol) of styrene oxide, 84 μL (0.92 mmol) of aniline, and 10 mg of catalyst were mixed. Some experiments were also performed using ethanol as solvent. The mixture then underwent reacting for 24 h, during which aliquots were extracted at the intervals of 1, 3, 6, and 24 h, and analyzed using GC-FID to investigate the progress of the reaction. An error of 5% was determine for the conversion of styrene oxide. The products obtained were identified by GC-MS.

## 3 Results and discussion

### 3.1 Preparation and characterization of the catalysts

The incorporation of the POM in the ZIF-8 framework was initially verified by FTIR spectroscopy, comparing the spectra of the composite materials with those of the POM and the respective solid support (Figure 1). POMs, particularly the Keggin-type structures, typically exhibit four prominent vibrational bands in the infrared range (Neves et al., 2013; Sampurnam et al., 2019). The FTIR spectra of both PMo_12_@ZIF-8 composites prepared by impregnation and *in-situ*, reveal the characteristic bands of the POM, namely, *ν*_as_ (P–O) at 1,057 cm^−1^, *ν*_as_ (Mo-O) at 959 cm^−1^, *ν*_as_ (Mo–O_b_–Mo) at 756 cm^−1^ and *ν*_as_ (Mo–O_c_–Mo) at 885 cm^−1^ (Figure 1, inserted highlight) (Mallik et al., 2006; Murugan et al., 2003; Neves et al., 2013; Zhai and Li, 2019). The ZIF-8 material displayed the anticipated peaks which can be ascribed to the stretching band ν (Zn–N) at 422 cm^−1^ (Fernandes et al., 2022; Hu et al., 2022; Zhang et al., 2018). This observation confirms the existence of ZnN_4_ clusters within the material. Additionally, a peak corresponds to C–H bending vibrations δ (C–H) at 760 cm^−1^ and another vibration is detected that arises from C–N bending motions at 994 cm^−1^. Furthermore, a peak observed at 1144 cm^−1^ can potentially be attributed to aromatic C–N stretching vibrations (Fernandes et al., 2022; Zhang et al., 2018). Lastly, a distinct absorption band appeared at 1,587 cm^−1^. This is associated to C=N stretching vibrations (Zhang et al., 2018). In the case of PMo_12_@ZIF-8 (imp and is samples), the main characteristic spectral bands related to the host ZIF-8 and the guest PMo_12_ were observed (Figure 1). The presence of Zn–N bond stretching vibrations was confirmed by a band at 422 cm^−1^ (Fernandes et al., 2022; Hu et al., 2022; Zhang et al., 2018). Additionally, C–H bending vibrations were detected at 759 cm^−1^, while C–N bending gave rise to a band at 994 cm^−1^ (Hu et al., 2022; Zhang et al., 2018). The aromatic C–N stretching vibration yielded an absorption peak at 1144 cm^−1^, whereas the C=N stretching vibration manifested as a band at 1583 cm^−1^. The peak observed at 870 cm^−1^ corresponds to Mo-O_b_-Mo bonding from PMo_12_ active center, while the one at 940 cm^−1^ may correspond to Mo=O_d_ vibrating mode (Mallik et al., 2006; Murugan et al., 2003). Finally, a very small peak is assigned to ν_as_ (P–O_a_) at 1069 cm^−1^.

**FIGURE 1:**
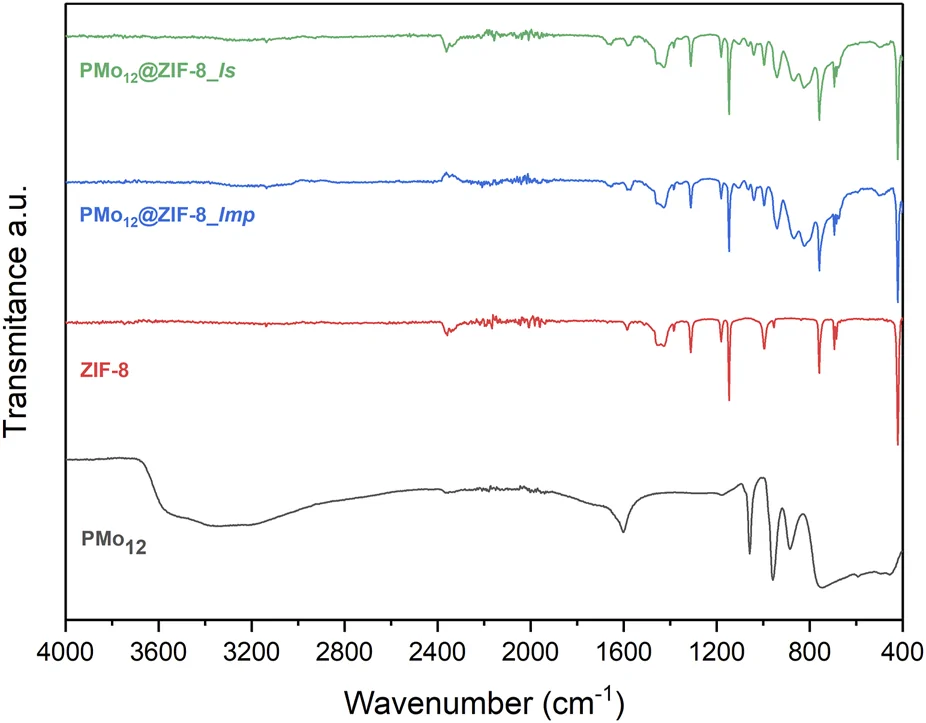
FTIR spectra of ZIF-8, PMo_12_ and PMo_12_@ZIF-8 composites, obtained by impregnation (imp) and *in-situ* (is) approach.

The ZIF-8 framework was subjected to powder X-ray diffraction analysis as a further test, and encouraging outcomes were achieved. The obtained diffractogram revealed the main characteristic reflections with 2θ values of 7.4°, 10.5°, 12.8°, 14.8°, 16.5° and 18.1° (Figure 2) which corresponded to the previously determined and reported structure of ZIF-8 (Md Nordin et al., 2017; Yao et al., 2013). The 2θ reflection bands positions for both composite materials revealed identical values, apart the PMo_12_@ZIF-8 prepared by impregnation presented an extra reflection band at 2θ of ~5° which can be likely attributed to a small modification in the electronic distribution of the ZIF-8 (support) caused by the guest PMo_12_ within the ZIF-8 framework. In fact, the powder X-ray diffraction results confirm that the introduction of POM did not lead to significant alterations in the overall crystalline structure of ZIF-8, suggesting a good dispersion of the POM within the ZIF-8 framework.

**FIGURE 2:**
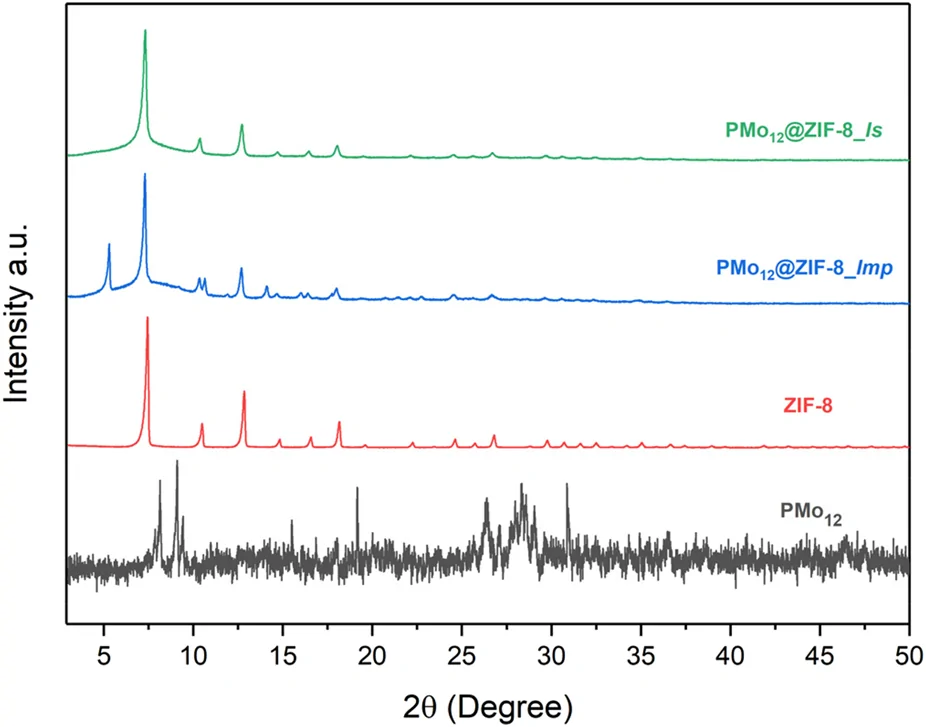
Powder X-ray diffractogram of the ZIF-8 and ZIF-8 composite materials, prepared by impregnation and *in-situ* approach.

The SEM technique was used to examine the morphologies of the materials, employing various approximations. During this analysis, the attention was focused exclusively on the three materials: the pristine ZIF-8 framework, and the composites PMo_12_@ZIF-8_*imp* and PMo_12_@ZIF-8_*is* prepared by impregnation and *in-situ* approach, respectively (Figure 3). When observing the ZIF-8 samples, it was evident that they possessed highly uniform dodecahedron shapes ranging in size from 460 to 540 nm. Conversely, a more irregular morphology with particle aggregation was noticed in the case of PMo_12_@ZIF-8 composites, suggesting that the incorporating of PMo_12_ into the framework had an impact on its overall structure and shape. Additionally, these particles showed smaller regular sizes and present considerably smaller sizes.

**FIGURE 3:**
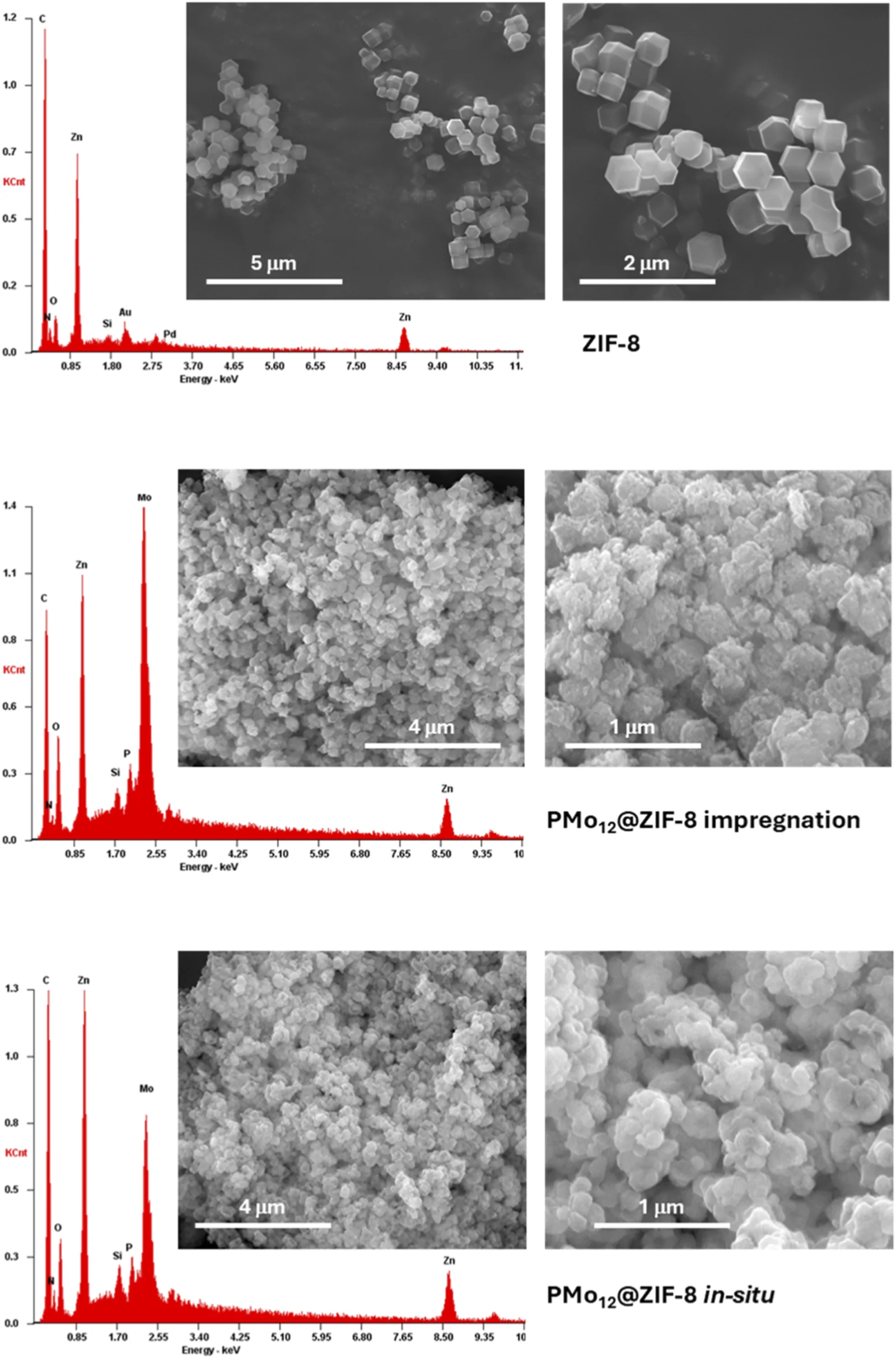
SEM images and respective EDS spectra of ZIF-8 Framework (top), PMo_12_@ZIF-8_*imp* (medium) and PMo_12_@ZIF-8_*is* (bottom) prepared by impregnation and *in-situ* approach, respectively.

The EDS analysis of the ZIF-8 structure revealed the presence of Zinc (Zn), Carbon (C), and Nitrogen (N), as expected (Figure 3). Furthermore, when analyzing the two composites PMo_12_@ZIF-8, it was confirmed that apart from Zn, C, and N elements found in ZIF-8, Phosphorus (P) and Molybdenum (Mo) were also detected. This detection confirms the presence of PMo_12_ active center in both the composite structure, PMo_12_@ZIF-8_*imp* and PMo_12_@ZIF-8_*is*.

In order to determine the quantity of PMo_12_ in the composite materials, i.e., the loading of the active center, an ICP analysis of both the composite materials PMo_12_@ZIF-8 was conducted. The *in-situ* composite (PMo_12_@ZIF-8_*is*) demonstrated a Mo loading of 171.6 g per kg of composite, corresponding to 149 μmol of PMo_12_ per gram of PMo_12_@ZIF-8_*is*, for the composite material prepared by the impregnation method, the amount of Mo in PMo_12_@ZIF-8_*imp* was found to be 223.8 g of Mo per Kg of composite (the loading of PMo_12_ corresponds to 194 μmol per gram of PMo_12_@ZIF-8_*imp*).

N_2_ adsorption-desorption isotherms were recorded at 77 K, and Type I isotherm are observed for both PMo_12_@ZIF-8 composites, which is the signature curve for purely microporous materials (Figure 4). Compared with the results obtained in the literature for the pristine ZIF-8, this exhibits a high specific surface area (1120 m^2^ g^−1^) with a total pore volume of 0.69 cm^3^ g^−1^ (Abdelkhalek et al., 2024), remarkable encapsulation of PMo_12_ using both methodologies (impregnation and *in-situ*) within ZIF-8 cavities preserved the microporous feature of the isotherm, and decreased the surface area to 453 m^2^ g^−1^ and 672 m^2^ g^−1^ for the impregnated and *in-situ* composite, respectively. Also, a significative reduction in the total pore volume is observed for both composites (0.25 cm^3^ g^−1^ for PMo_12_@ZIF-8_*imp* and 0.36 cm^3^ g^−1^ for PMo_12_@ZIF-8_*is)* compared to the pristine ZIF-8, confirming the successful encapsulation of the PMo_12_ units within the ZIF-8 framework. The difference observed betweenPMo_12_@ZIF-8_*imp* and PMo_12_@ZIF-8_*is* may be related to the loading of PMo_12_ encapsulated.

**FIGURE 4:**
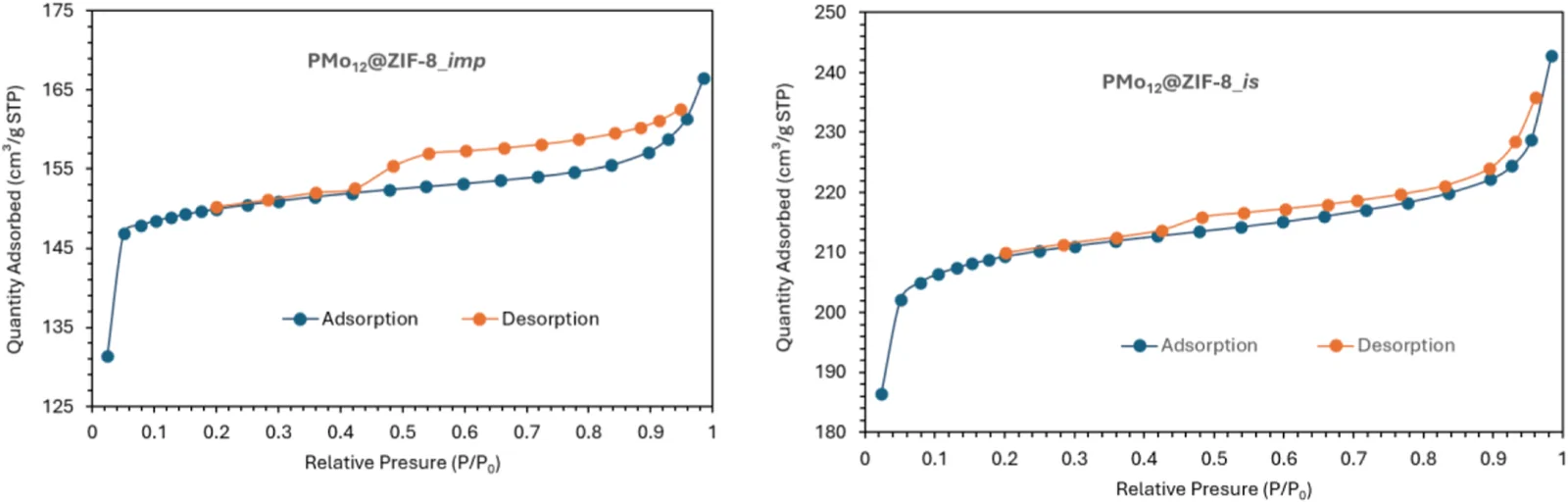
N_2_ adsorption-desorption isotherms of the PMo_12_@ZIF-8 composites prepared by impregnation (PMo_12_@ZIF-8_*imp*) and *in-situ* procedures (PMo_12_@ZIF-8_*is*).

### 3.2 Catalytic studies

The materials prepared and characterized previously were tested as heterogeneous catalysts of the ring-opening reaction of styrene oxide (ROSO) with aniline, under specific conditions. The reactions were conducted at 70 °C for a period of 24 h, under a solvent-free system and using ethanol as solvent. Initially, a blank experiment was performed in the absence of catalyst to establish baseline conditions and confirm that the reaction does not proceed efficiently without catalytic assistance. In this case, a conversion of only 24% was obtained after 24 h. The catalytic behavior of the pristine ZIF-8 framework was also evaluated; however, this was like obtained without catalyst (Supplementary Table S1), with a conversion no higher than 24% after 24 h of reaction. Subsequently, the heterogeneous catalytic performance of the ZIF-8-based composites PMo_12_@ZIF-8_*imp* and PMo_12_@ZIF-8_*is*, prepared by impregnation and *in-situ* procedures, respectively, was investigated. These catalytic studies were performed under a solvent-free system and using ethanol as solvent. The results were comparable in both conditions; however, slightly higher conversions were achieved using the composite prepared by impregnation PMo_12_@ZIF-8_*imp* (Figure 5). Using this catalyst approximately 81% of conversion after 6 h and 97% after 24 h were achieved, when 0.5 mL of ethanol was used. However, under a solvent-free system similar conversion was achieved after 24 h, and a remarkable increase of conversion could be observed after the 3 h. This enhanced performance is likely attributed to the successful immobilization of the active center phosphomolybdic acid PMo_12_ and its relatively higher loading amount than the obtained for PMo_12_@ZIF-8_*is*. This last composite prepared *in-situ* exhibited slightly lower catalytic efficiency, particularly at reaction times below 24 h, but reaching a conversion of 98**%** after 24 h. This reduced activity can be correlated with the lower PMo_12_ loading typically obtained by the *in-situ* synthesis route. The average TOF values (after 24 h of reaction) calculated for both composites resulted in 29.94 h^−1^ for PMo_12_@ZIF-8_*is*, and 22.95 h^−1^ for PMo_12_@ZIF-8_*imp*.

**FIGURE 5:**
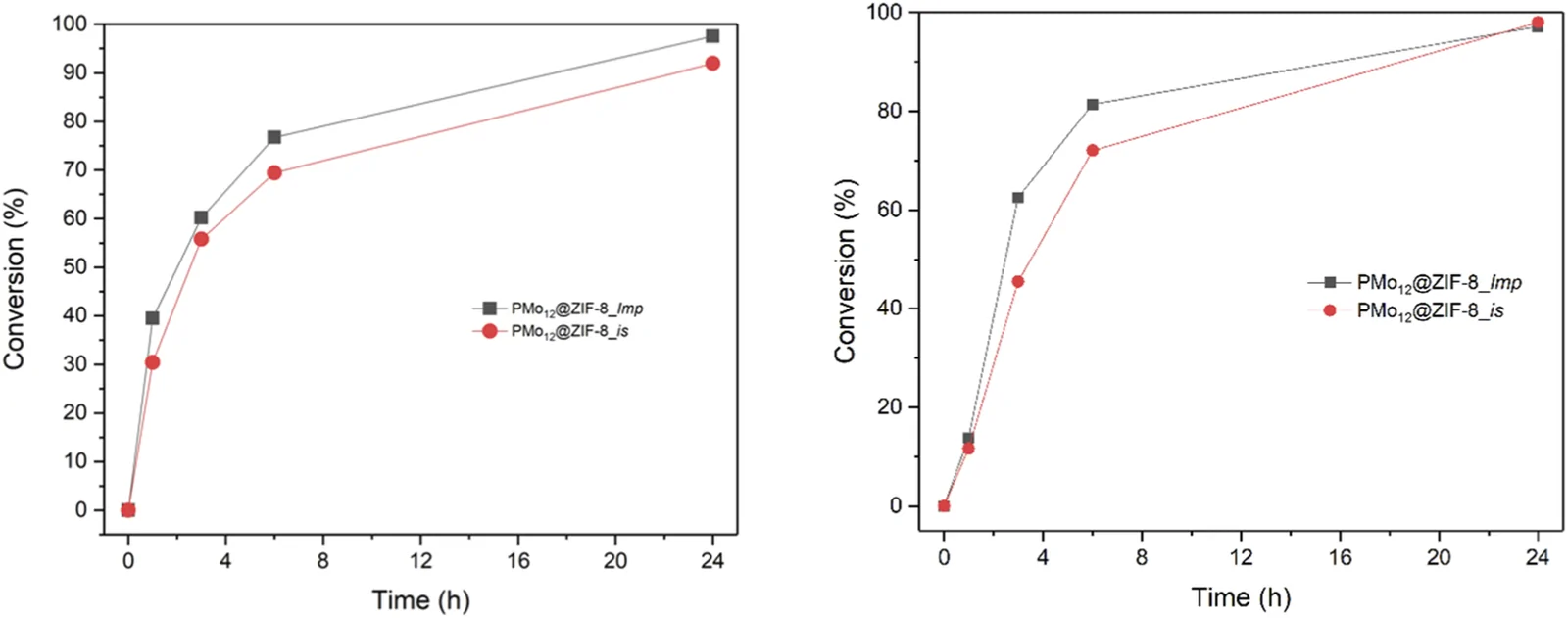
Kinetic profile for the ROSO reaction with aniline, using PMo_12_@ZIF-8_*imp* and PMo_12_@ZIF-8_*is* composites (10 mg), at 70 °C, using 0.5 mL of ethanol as solvent (left side) and under a solvent-free system (right side).

According with the high catalytic efficiency of the PMo_12_@ZIF-8 composites, the influence of temperature and the presence of ethanol as solvent was investigated. The ROSO reaction was performed varying the temperature between 25 °C and 70 °C (Figure 6), and it is possible to observe that the conversion decreased drastically when 25 °C was used instead of the previous 70 °C. Furthermore, the presence of the solvent as ethanol in the ROSO reaction seemed do not have an important influence, mainly after the 3 h of reaction. The kinetic profile obtained between the Solvent-Free system (S.F in Figure 6) and the one using 0.5 mL of ethanol, were similar mainly after the 4 h of reaction, when the conversion was higher than 60%, achieving in both cases a final conversion of 98% after the 24 h. Additionally, it was observed an identical catalyst selectivity in the presence and absence of ethanol. According to these results, future experiments were carried out at 60 °C and using the solvent-free system, making the ROSO reaction even more sustainable.

**FIGURE 6:**
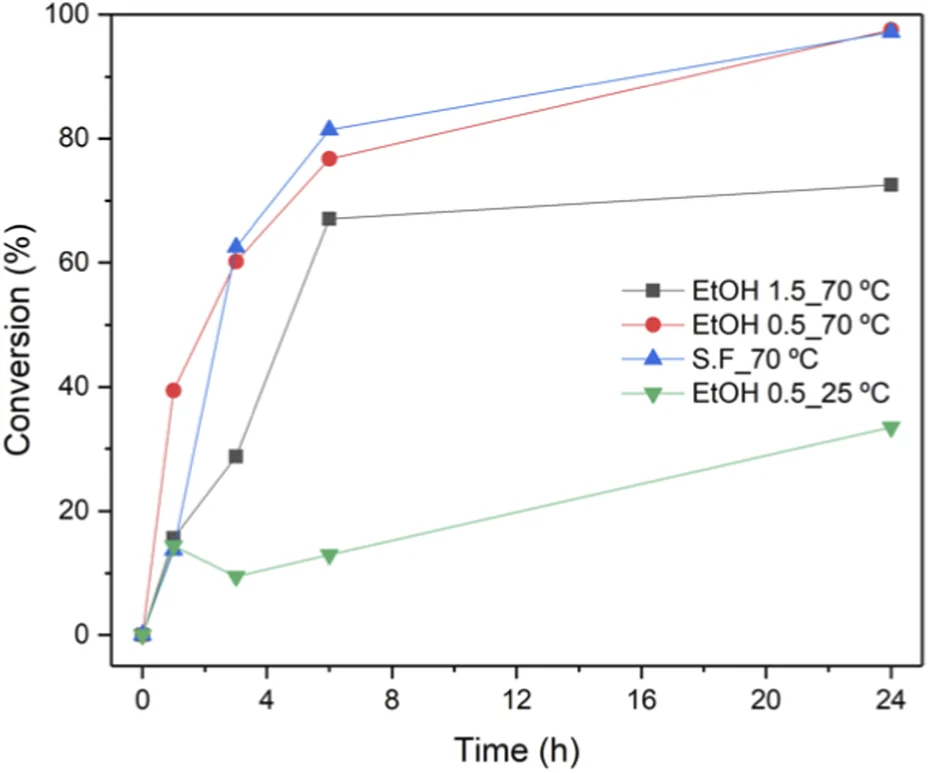
Kinetic profile for the ROSO reaction with aniline, using PMo_12_@ZIF-8_*imp* catalyst (10 mg), varying the temperature (25 °C and 70 °C), and using different volumes of ethanol as solvent (0.5 and 1.5 mL) and also under a Solvent-Free (S.F) system.

Under the solvent-free system and using the ethanol solvent, the products obtained were identified by GC-MS analysis. Results are presented in Supplementary Figure S1 for the mass spectra resulting from GC-MS analysis to the main product obtained, that could be identified as 2-Amino-1,2-diphenylethanol by the similarity with the simulated spectrum (Supplementary Figure S1). Identical results were obtained previously (Ferreira et al., 2024). Supplementary Figure S2 displays the ^1^H NMR spectrum obtained after the analysis of reaction after 24 h. Peaks for -NH_2_ and -OH protons, along with multiplets for aromatic protons are presented in the region between 6.5 and 7.5 ppm. Furthermore, peaks are presented near 4.1 ppm for the methine adjacent to the amine, and around 4.5 ppm signals can be attributed for protons of the methine adjacent to the alcohol. Peaks presented between 2.0 and 3.0 ppm correspond to the unreacted acetone and glycerol still presented.

Using both PMo_12_@ZIF-8 composites, higher selectivity was observed for amino derivate. Table 1 presents the conversion and selectivity obtained for ROSO reaction with aniline at 70 °C, catalyzed by both PMo_12_@ZIF-8 composites, under the solvent-free system. The main product was the 2-Amino-1,2-diphenylethanol (Figure 7), obtained with selectivity between 87% and 86%, depending on the catalyst. The Amino diphenylmethane was obtained with a selectivity between 13% and 15%. It is possible to verify a similar catalytic behavior between both composites, for the conversion and also for the selectivity results. This similarity may indicate that the active centers must be immobilized identically in both composites although the distinct impregnation and *in-situ* preparation procedures. The accessibility of the active Lewis PMo_12_ catalytic center and the diffusion of reactant though the pores of these heterogeneous catalysts based in ZIF-8 must be similar in both composites. The mechanism involves initially the interaction of the Lewis catalyst with the epoxide that activates the epoxide by dramatically increasing the electrophilicity of this group. This significantly weakens and polarizes the C-O bonds, making the adjacent benzylic carbons highly electrophilic. The amine nucleophile attacks one of the activated benzylic carbons, causing the carbon-oxygen bond to break. The attack follows an S_N_2-like pathway. The oxygen-metal bond breaks as the ring opens, leaving an alkoxide complexed to the metal, while the incoming amine carries a positive NH_3_^+^. An intermolecular proton transfer occurs: the alkoxide takes a proton from the quaternary ammonium group. This proton shift neutralizes the molecule, forming the stable 1,2-amino alcohol product and releasing the Lewis acid catalyst back into the catalytic cycle to bind a new epoxide molecule (Mai and Schneider, 2007; Wosińska-Hrydczuk and Skarżewski, 2019).

**TABLE 1:** Conversion and selectivity data obtained for ROSO reaction with aniline after 24 h, catalyzed by PMo_12_@ZIF-8 composites under a solvent-free system, at 70 °C.

Catalysts	Conversions (%)	Selectivity (%)	
		2-Amino-1,2-diphenylethanol	Aminodiphenylmethane
ZIF-8@PMo_12__*imp*	97.2	87.0	13.0
ZIF-8@PMo_12__*is*	98.0	85.5	14.5

**FIGURE 7:**
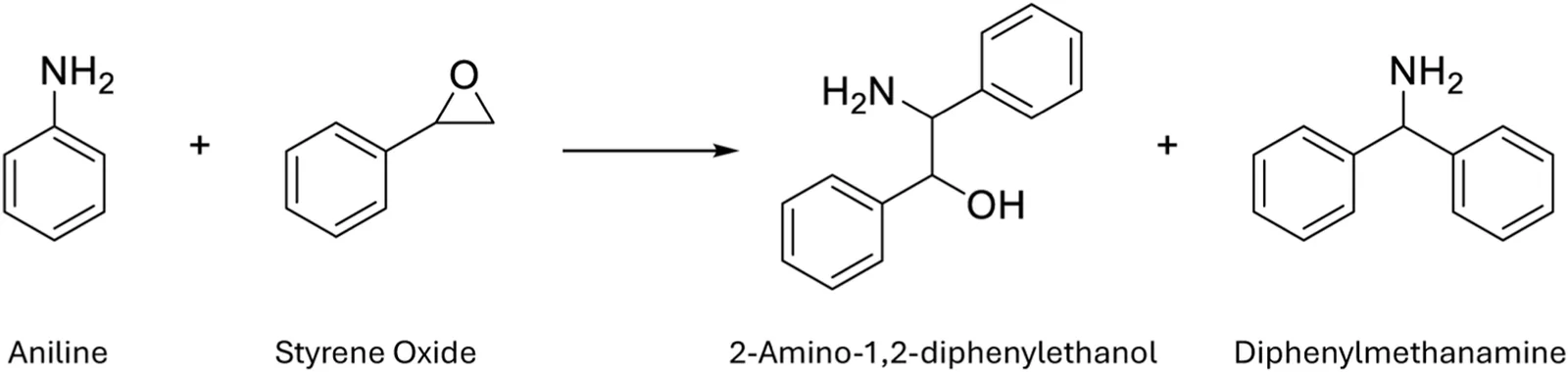
Illustration of the ROSO reaction with aniline when catalyzed by PMo_12_@ZIF-8 composites at 70 °C, showing two main products after 24 h.

### 3.3 Catalyst recycling and stability

The capacity of recycling the heterogeneous catalysts ZIF-8 composites was investigated to test their stability when used under consecutive ROSO reactions. In these studies, the reaction conditions were maintained between the different cycles, performed at 70 °C and under the solvent-free system. The solid catalyst after the 24 h of reaction was removed from reactional solution, washed carefully with several cycles of ethanol and dried before used in a novel reaction. The results obtained are presented in Figure 8 (kinetic profiles for consecutive reactions are presented in Supplementary Figure S3).

**FIGURE 8:**
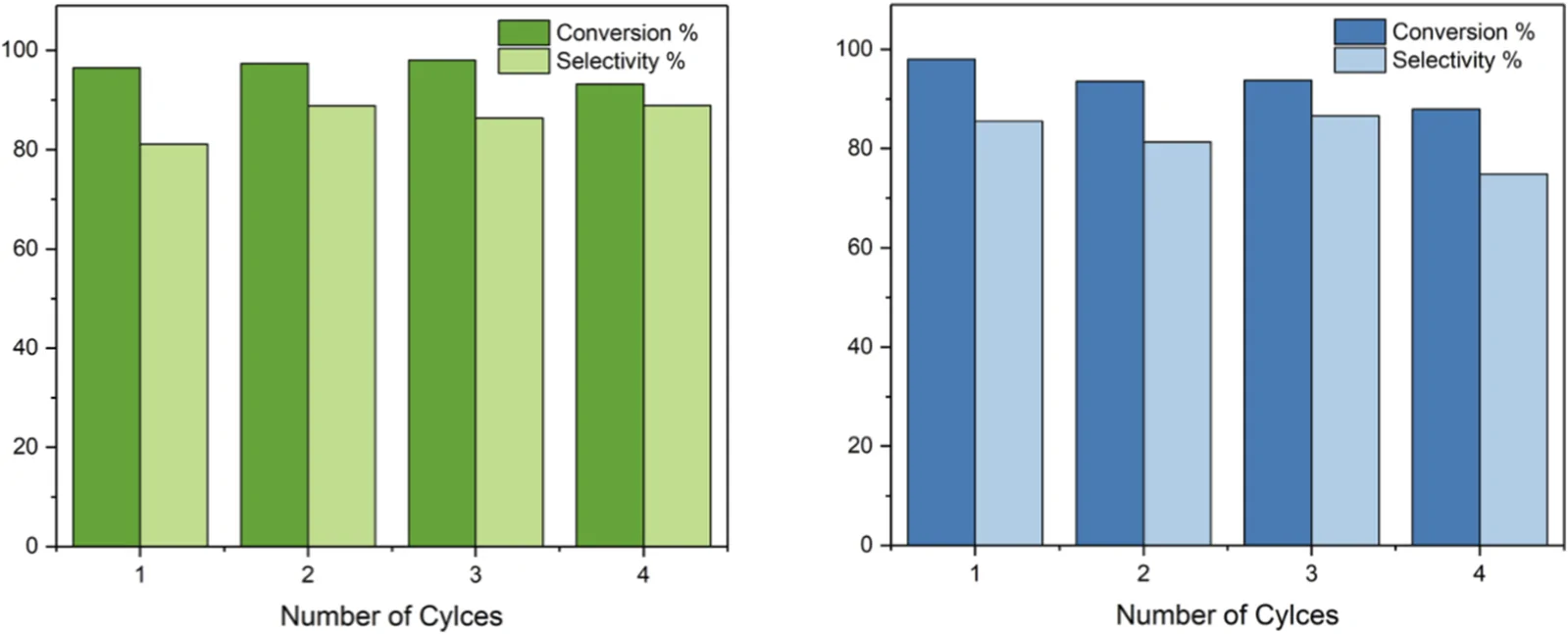
Conversion and selectivity data obtained for four consecutive ROSO reactions with aniline, using the catalysts (left) PMo_12_@ZIF-8_imp and (right) PMo_12_@ZIF-8_is, at 70 °C, under a solvent-free system.

Notably, PMo_12_@ZIF-8_*imp* prepared by impregnation procedure was found to maintain its catalytic performance with conversion results always above 90% during all the recycling process, without losing conversion or selectivity. On the other hand, the heterogeneous catalyst prepared by *in-situ* approach exhibited high catalytic efficiency in the first cycle, followed by a slight decrease in activity in subsequent cycles. The conversion was decreasing between consecutive cycles, what also occurred for the selectivity of this catalyst. The minor deactivation of this *in-situ* prepared catalyst must be due to some partial loss of accessibility of active centers PMo_12_ between cycles, probably caused by pore blockage or by a minor PMo_12_ leaching after the first reaction cycle.

To investigate the structural stability of the catalysts after catalytic use, these were characterized by several techniques after. The FT-IR spectroscopy and powder XRD analysis were performed of the catalyst after the four recycling cycles (Figure 9). The FTIR spectrum was found to be identical to that of the previous used PMo_12_@ZIF-8 composites, where it was possible to identified the main bands attributed to the pristine ZIF-8 framework, and also the characteristic bands *ν*_as_ (P–O) at 1057 cm^−1^, *ν*_as_ (Mo-O), *ν*_as_ (Mo–O_b_–Mo) and *ν*_as_ (Mo–O_c_–Mo) attributed to the PMo_12_. By comparing the PXRD patterns of the recovered catalyst after four cycles and the previous used in catalysis, it is possible to confirm that the global crystalline structure of the ZIF-8 was maintained, since the position of practically all the reflection/peaks is maintained. However, some modifications in the relative intensity of the peaks were further verified (intensity of peaks at 2θ greater that 10° increase considerably relative to the peak around 7.5°), pointing to changes in the internal electronic distribution in the ZIF-8 support caused by the catalytic reaction, in both the composite materials, PMo_12_@ZIF-8_*imp* and PMo_12_@ZIF-8_*is*.

**FIGURE 9:**
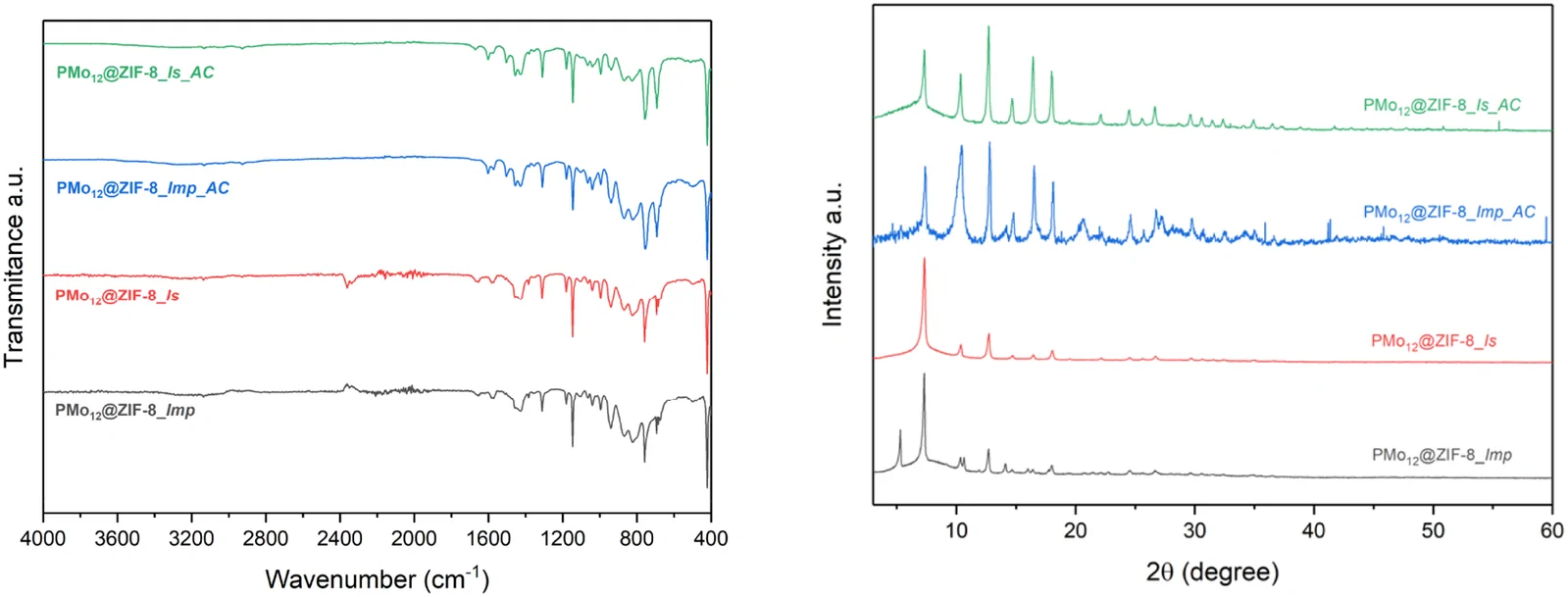
FT-IR spectra (on left) and Powder XRD patterns (on right) of the ZIF-8 based composites before and after catalytic application (sample recovered after four consecutive ROSO reaction cycles; AC stands for after catalytic use).

SEM images of the ZIF-8 based composites after four catalytic cycles did not reveal any significant morphological changes when compared to the fresh materials, indicating that the structural integrity of the composites was preserved during the catalytic process (Supplementary Figure S4). EDS analysis showed that the chemical composition of ZIF-8-based impregnation composite remained essentially unchanged after catalysis. For the PMo_12_@ZIF-8_*imp*, the Mo/Zn ratio varied slightly from 0.87 (fresh catalyst) to 0.73 (after catalysis), while for PMo_12_@ZIF-8_*is*, this ratio changed from 0.40 to 0.45 after the catalytic reaction. Moreover, the liquid solution that was separated from the solid catalyst after the fourth cycle was analyzed by ^31^P NMR. No peaks were observed, confirming the absence of leaching of the PMo_12_ species from the PMo_12_@ZIF-8 based composites (Supplementary Figure S5). Therefore, the slight decrease of activity observed for the *in-situ* catalyst after the first cycle may not be attributed to the loss of active centers, but probably due to the structural modification of the support (causing some loss of accessibility to the active centers originating some pore blockage). Even that, the catalysts PMo_12_@ZIF-8 based composites demonstrated to be catalytically active and reusable for the ring opening epoxidation of aniline and styrene oxide reaction under a solvent-free system, producing excellent selectivity toward 2-Amino-1,2-diphenylethanol.

### 3.4 Comparison with literature results

Table 2 display the few examples that can be find in the literature using MOF structures as heterogeneous catalysts for the ring-opening reaction of epoxides with aniline. Jiang et al. in 2008 was the first reporting in this area. In this work, Cu-MOF catalyst was used at room temperature under a solvent-free system, achiving only a conversion of 32% of cyclohexene oxide after 4 h, needing to use a high excess of aniline (21 mmol, instead of 0.9 mmol used in this work) (Jiang et al., 2008). The low catalytic results was due to diffusion limitations in the micropores of Cu-MOF. Two years later, Garcia et al. used Fe-BTC MOF as catalyst for the ring-opening of styrene oxide using acetonitrile as solvent (10 mL). A conversion of 72% was obtained after 24 h, in the presence of substrate/catalyst ratio of 410 (Dhakshinamoorthy et al., 2010). Some years later (2018), the same Fe-BTC MOF and the cupper Cu_3_(BTC)_2_ catalysts were used for the styrene oxide reaction with aniline, without using a solvent; however a high amount of catalyst needed to be used to achieve a moderate conversion result of epoxide (Anbu and Dhakshinamoorthy, 2018). The same reactions have been investigated by Kumar et al. using [Co^3+^-Ln^3+^] heterodimetallic one-dimensional zigzag coordination polymers as heterogeneous catalysts (Kumar et al., 2010). β-amino alcohols were obtained with high yields using near equimolar ratio of substrates and aniline, at room temperature and without solvent needed. This dimetallic catalyst was recycled during three cycles and some loss of activity was found. In 2017, our research group used the MIL-101(Fe) as heterogeneous catalyst for the ring-opening reaction of cyclohexene oxide with aniline (Barbosa et al., 2017). Under low sustainable conditions (acetonitrile used as solvent) a high amount of catalyst was needed to achieve high conversion. To analyse the importance of Fe in the styrene oxide aminolysis reaction, the MIL-101(Fe)-NH_2_ was used as support to incorporate a iron-monosubstituted polyoxometalate [PW_11_Fe(H_2_O)O_39_]^5-^ (abreviatted as PW_11_Fe) (Julião et al., 2017). This work was the first to use a polyoxometalate@MOF composite as heterogeneous catalyst for the ring-opening reaction of epoxide with aniline. In this study, the catalyst performance increased drastically and pratically complete styrene oxide conversion was obtained after 1 h, instead of 22% obtained the support MIL-101(Fe)-NH_2_. Our research group continue to investigate other families of MOFs as catalysts for this reaction. Recently, MOF-808 and UiO-66-NH_2_ were tested and high catalytic efficiency was obtained using low amount of solid MOF catalysts, still using ethanol as solvent (Ferreira et al., 2024). In this work advanced heterogeneous catalysts based in PMo_12_@ZIF-8 showed an improvement of catalytic results for the open-ringing of styrene oxide with aniline, since higher conversions were observed under a solvent-free system. The contribution for this improvement was due to the strategic immobilization of the highly active PMo_12_ into ZIF-8 framework.

**TABLE 2:** Reported works for the ring-opening reaction of epoxides (stryrene oxide and cyclohexene oxide) with aniline catalysed by MOF based materials.

Epoxide	Catalyst	Catalyst amount	Epoxide/Aniline (mmol)	Solvent	T (°C)	Time (h)	Conv. (%)	Refs.
CyclohexOx	Cu-MOF	0.11 mmol	1/21	no	rt	4	32	Jiang et al. (2008)
	[Co^3+^-Ln^3+^]^a^	49 μmol	0.98/1.18	No	rt	4	98	Kumar et al. (2010)
	MOF-808	1 μmol	1/0.9	EtOH	70	24	88	Ferreira et al. (2024)
	MIL-101(Fe)	55 μmol	1/0.9	MeCN	80	24	87	Barbosa et al. (2017)
	MIL-101(Fe)-NH_2_	55 μmol	1/0.9	MeCN	80	24	86	Barbosa et al. (2017)
StyrOx	MOF-808	1 μmol	1/0.9	EtOH	70	6	83	Ferreira et al. (2024)
	UiO-66-NH_2_	1 μmol	1/0.9	EtOH	70	24	88	Ferreira et al. (2024)
	MIL-101(Cr)	50 μmol	1/0.9	MeCN	80	5	0	Julião et al. (2017)
	Cu_3_(BTC)_2_	25 mg	0.3/0.25	No	60	24	75	Anbu and Dhakshinamoorthy (2018)
	Fe-BTC	25 mg	0.3/0.25	no	60	24	77	Anbu and Dhakshinamoorthy (2018)
	Fe-BTC	150 mg	41.6/41.6	MeCN	80	24	72	Dhakshinamoorthy et al. (2010)
	[Co^3+^-Ln^3+^]^a^	44 μmol	0.87/1	No	rt	4	84	Kumar et al. (2010)
	PW_11_Fe@MIL-101(Fe)	50 μmol	1/0.9	MeCN	80	1	97	Julião et al. (2017)
	PMo_12_@ZIF-8_is	1 μmol	1/0.9	No	70	24	98	Here
	PMo_12_@ZIF-8_imp	1 μmol	1/0.9	No	70	24	97.2	Here

^a^ The catalyst [Co3+-Ln3+] is a coordination polymer with Ln = Eu or Tb; rt = room temperature.

## 4 Conclusion

In this work was successfully prepared ZIF-8 based catalytic materials by effective immobilization of phosphomolybdic acid as active center, PMo_12_, within the suitable cavities of the MOF. Two different immobilization strategies were investigated: impregnation and *in-situ* incorporation. The successful synthesis of both the composite and support material was confirmed through various techniques, such as FTIR, PXRD, SEM, EDS and ICP-OES analyses. FTIR spectroscopy verified the presence of PMo_12_ in the composite materials, while PXRD confirmed the preservation of the crystalline ZIF-8 framework after functionalization with PMo_12_. SEM analysis provided information on particle morphology, and EDS confirmed the elemental composition, including Zn from the ZIF-8 framework and Mo from POM active species. ICP-OES quantification demonstrated effective loadings of PMo_12_ in the ZIF-8 structure, confirming the success of the immobilization procedures.

The catalytic performance of the PMo_12_@ZIF-8 composites was evaluated in the ring-opening epoxidation of styrene oxide with aniline (ROSO), under both solvent and solvent-free conditions. Higher conversions were obtained under solvent-free conditions, where both catalysts demonstrated good activity, achieving 97% and 98% conversion with 87% and 85% selectivity 2-Amino-1,2-diphenylethanol after 24 h, for the impregnation and *in-situ* methods, respectively. Further, the recycling studies revealed that both PMo_12_@ZIF-8 catalysts could be reused for at least four consecutive cycles. The impregnated material maintained catalytic activity and high selectivity, while the *in-situ* composite showed a slight decrease in conversion during recycling process. Importantly, both catalysts retained their structural integrity after recycling, demonstrating their potential as robust heterogeneous catalytic systems.

## Data Availability

The original contributions presented in the study are included in the article/Supplementary Material, further inquiries can be directed to the corresponding author.
