# Supplementary material for: Active porous ZIF-8 based hybrid catalysts for solvent-free reactions
Source: Front Chem. 2026 Sep 17;14:1894924. doi: 10.3389/fchem.2026.1894924 (PMC13627323; doi:10.3389/fchem.2026.1894924)
Supplement: Supplementary file 1 [file DataSheet1.docx]

Active porous ZIF-8 based hybrid catalysts for solvent-free reactions

**César D. Sampaio^1^, Luis Dias^1^, Isabel Santos-Vieira^2^, Eulália Pereira^1^,
Luís Cunha-Silva^1,3^, Fátima Mirante^1^, Salete S. Balula^1,4*^**

^1^ LAQV/REQUIMTE—Laboratório Associado para a Química Verde e Departamento de Química e Bioquímica & Faculdade de Ciências, Universidade do Porto, 4169-007 Porto, Portugal

^2^ CICECO – Aveiro Institute of Materials, Department of Chemistry, University of Aveiro, 3810-193 Aveiro, Portugal

^3^ LNEG - Laboratório Nacional de Energia e Geologia, I.P., Estrada do Paço do Lumiar, 1649-038 Lisboa, Portugal

^4^LAQV / REQUIMTE & Department of Chemistry, Nova School of Science and Technology, Nova University of Lisbon, 2829-516 Monte da Caparica, Portugal

*Corresponding author: sbalula@fc.up.pt (S.S.B.)

**Table S1.** Conversion and selectivity data obtained for ROSO reaction using blank conditions (without catalyst), using the support ZIF-8 and the homogeneous active center PMo_12_.

| Catalysts | Conversion % | Selectivity % | |
| --- | --- | --- | --- |
|  |  | 2-Amino-1,2-diphenylethanol | Amino diphenylmethane |
| Blank | 24,3 | 87,6 | 12,4 |
| ZIF-8 | 19,5 | 69,5 | 30,5 |
| PMo_12_ | 91,5 | 72,6 | 27,4 |

**Figure S1.** Mass spectra obtained from GC-MS analysis from ROSO reaction with aniline, using the catalysts PMo_12_@ZIF-8_imp at 70 °C, under a solvent-free system (top is the experimental analysis and bellow is the simulated spectrum for 2-Amino-1,2-diphenylethanol).

**Figure S2.** ^1^H NMR analysis obtained from ROSO reaction with aniline, using the catalysts PMo_12_@ZIF-8_imp at 70 °C, under a solvent-free system.

**B**

**A**

**Figure S3.** Conversion data and selectivity data obtained for the ROSO reaction with aniline, using the catalysts (a) PMo_12_@ZIF-8_*imp* and (b) PMo_12_@ZIF-8_*is*, at 70 °C, using EtOH as solvent.

**Figure S4.** SEM obtained for PMo_12_@ZIF-8_*imp* (top) and PMo_12_@ZIF-8_*is* (bottom) after four cycles of reutilization.

**A**

**B**

**Figure S5.** ^31^P NMR spectra obtained from the reactional solution at the end of the epoxidation reaction catalyzed by A - PMo_12_@ZIF-8_*imp* and B - PMo_12_@ZIF-8_*is* after 4^th^ consecutive cycles.
